# Patients with (familial) atrial fibrillation: take off the sweater

**DOI:** 10.1007/s12471-024-01891-7

**Published:** 2024-08-16

**Authors:** Andrea Bochem, Lucas V. A. Boersma, Saskia N. van der Crabben

**Affiliations:** 1 Department of Cardiology, St. Antonius Ziekenhuis, Nieuwegein, The Netherlands; 2 Department of Human Genetics, Amsterdam University Medical Centre, Amsterdam, The Netherlands

## Answer

The photo (Fig. 1 in the Question) shows hyperkeratosis and scaling of the skin. Because his maternal grandfather had the same skin abnormality and his mother was said to have dry skin, a clinical suspicion of X‑linked recessive ichthyosis arose [1]. This inherited skin disease is caused by deficiency of the steroid sulfatase (STS) enzyme, most often by a small deletion on the X‑chromosome (at Xp22.31) spanning the STS gene. Indeed, additional chromosomal analysis (SNP array) in our patient revealed the (1.8 Mb) Xp22.31 deletion.

Recently it was reported that male STS deletion carriers show an increased incidence of sinus bradycardia, atrial fibrillation (AF) and atrial flutter (10.5% versus 2.7% in male controls), possibly by affecting circulating dehydroepiandrosterone sulfate levels [2, 3]. Although the pathophysiological background is still unclear and does not alter current treatment, this genetic diagnosis is important as it provides clarity for the patient and his family and could prevent further (unnecessary) diagnostics. Moreover, it enables cascade screening in the family and critical patient appraisal in case of cardiac complaints even in younger patients. In the relentless search for AF pathophysiology and treatment, STS function may provide a piece of the puzzle and form a potential target for future gene therapy. Upon questioning, the patient knew he had been diagnosed with ichthyosis. This was not recorded in the patient charts because it was not considered a medical concern (by the patient). Although this is indeed the case in most patients, we would advise inspecting the skin in cases of (familial) AF in (young) patients.
